# Sustainable Innovations in Stone Matrix Asphalt: Integrating Recycled Materials and Low-Emission Production

**DOI:** 10.3390/ma19050937

**Published:** 2026-02-28

**Authors:** Mutahar Al-Ammari, Ruikun Dong, Guobing Deng, Salman Abdullah

**Affiliations:** 1 School of Civil Engineering, Chongqing University, Chongqing 400045, China; l2400098@stu.cqu.edu.cn; 2 Department of Civil Engineering, Faculty of Engineering, Sana’a University, Sana’a 9671, Yemen; 3 Chongqing Transportation Engineering Quality Inspection Co., Ltd., Chongqing 400714, China

**Keywords:** Stone Matrix Asphalt (SMA), sustainable pavement, pavement durability, recycled materials, green pavement technology

## Abstract

**Highlights:**

**What are the main findings?**

- SMA’s strength relies on a stone-on-stone skeleton and fiber stabilization.
- Polymers and crumb rubber greatly enhance rutting and fatigue resistance.
- Nanomaterials improve moisture resistance and aging performance.
- Recycled materials boost sustainability without sacrificing key properties.
- Warm Mix Asphalt tech lowers production energy and emissions significantly.

**What are the implications of the main findings?**

- Allows tailored pavement design for local climate and traffic conditions.
- Promotes sustainability by reducing landfill waste and carbon footprint.
- Enables cooler, safer construction via Warm Mix Asphalt practices.
- Prioritizes research on nano-modifiers, renewable binders, and self-repairing pavements.
- Calls for updated mix design standards to include new materials.

**Abstract:**

Stone Matrix Asphalt (SMA) has emerged as a premier high-performance paving solution for critical infrastructure applications. Its distinctive skeleton structure, composed of coarse aggregates bound by a fiber-stabilized bituminous mastic, delivers exceptional mechanical performance, including superior resistance to rutting (≤3 mm after 10^6^ load cycles) and fatigue cracking (>500,000 cycles to failure). While proven in demanding service environments, research has increasingly focused on enhancing the sustainability of SMA through key innovations: (1) the incorporation of recycled materials, such as 30–40% Reclaimed Asphalt Pavement (RAP) and 0.3–0.5% waste tire textile fibers (WTTF); (2) the development of bio-based binders derived from renewable sources; and (3) the adoption of Warm-Mix Asphalt (WMA) technologies that reduce production temperatures by 20–30 °C. These advancements yield significant environmental benefits, including approximately 25% lower CO_2_ emissions and 15–20% reduced energy consumption compared to conventional SMA production. It is important to distinguish between these quantitatively demonstrated benefits, primarily from Life Cycle Assessment (LCA) studies of technologies like WMA and RAP, and the more qualitative sustainability claims associated with emerging materials like nanomaterials or novel bio-additives, which often lack comprehensive lifecycle inventories. Nevertheless, challenges persist, notably moisture susceptibility (manifesting as a 10–15% strength reduction after saturation) and uncertainties regarding the long-term performance of modified mixes. This review consequently identifies critical research priorities: optimizing mix designs with locally available materials to minimize transport emissions, employing nano-scale modifiers to enhance moisture resistance, and developing standardized lifecycle assessment protocols. Addressing these challenges is paramount to establishing SMA as a model sustainable pavement technology that robustly meets both structural performance benchmarks and ecological sustainability goals.

## 1. Introduction

SMA is a highly durable and reliable paving material, particularly suited for high-traffic zones and demanding engineering projects. Its unique composition and design make it perfect for highways, bridges, and airport runways, where strength, load-bearing capacity, and performance under extreme conditions are critical. SMA excels in resisting deformation, preventing rutting, and withstanding harsh weather, making it a preferred choice for global infrastructure projects. This article explores SMA’s key features, benefits, technological advancements, applications, composition, manufacturing processes, and future potential.

### Literature Review Statistics

**Figure 1:**
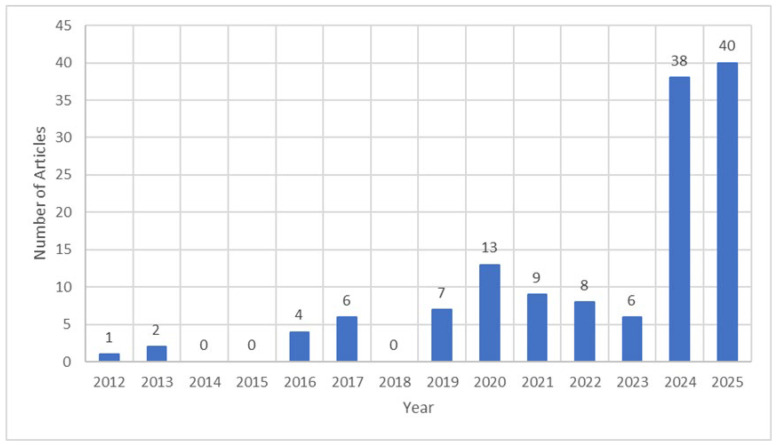
Number of published articles since 2012 to 2025. (Data retrieved from Scopus, Web of Science, and Google Scholar on, 23 March 2025).

The literature review explored three key databases: Scopus, Web of Science, and Google Scholar, which together provide the largest online repository for transportation engineering research. Figure 1 shows the annual publication trend from 2012 to 2025, with a total of 134 research papers published on this topic.

**Figure 2:**
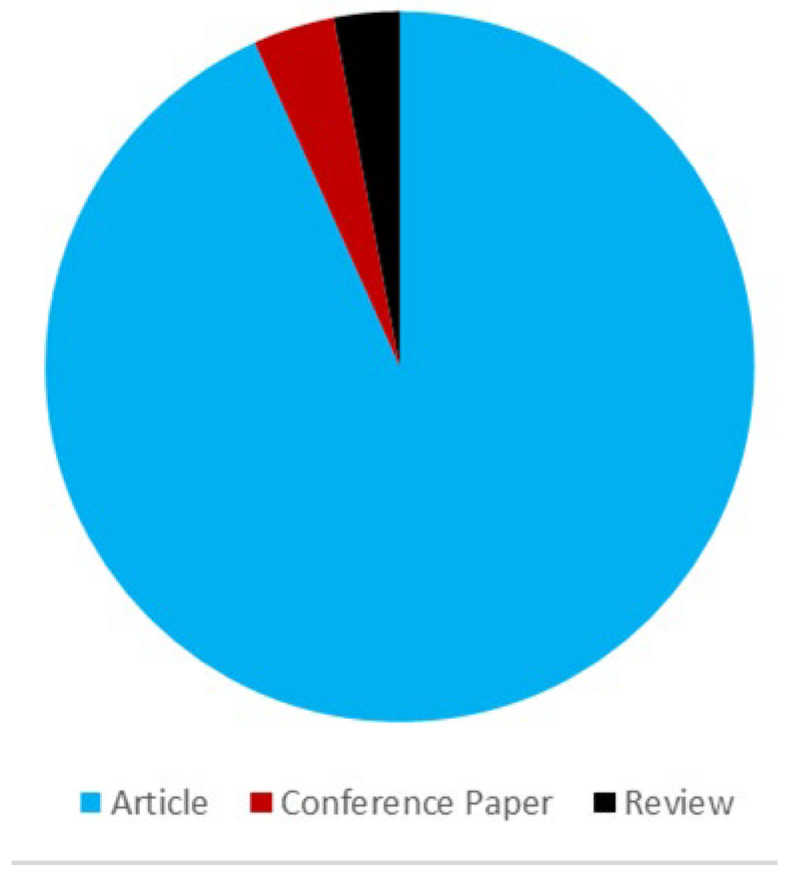
Document Type Distribution.

The distribution of document types across the corpus of 134 publications is visualized in Figure 2. The literature corpus is overwhelmingly dominated by Articles (*n* = 125, 93.3%), with a much smaller proportion of Conference Papers (*n* = 5, 3.7%) and Reviews (*n* = 4, 3.0%).

**Figure 3:**
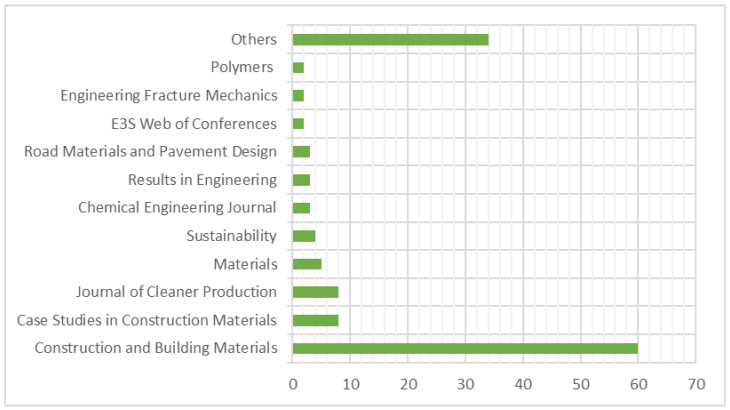
Journals published documents related to the topic.

Research on this topic appeared in 45 different journals, as depicted in Figure 3. The most significant contributor was Construction and Building Materials, representing 44.8% of all publications, while the Journal of Cleaner Production and Case Studies in Construction Materials followed closely with 6% of the total papers published on the subject.

**Figure 4:**
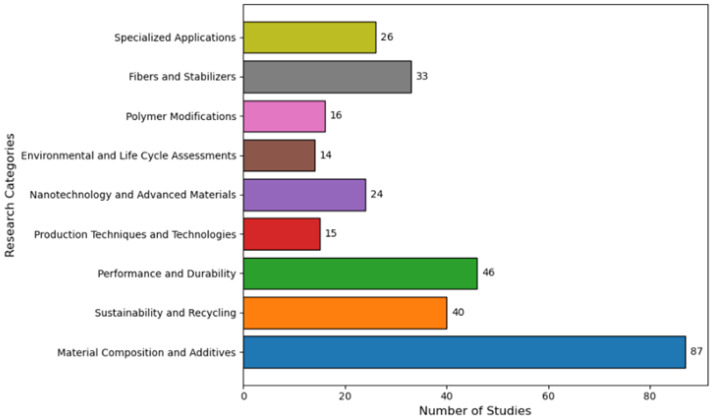
Primary research disciplines.

The analyzed research primarily focuses on engineering and materials science disciplines. Figure 4 displays the subject area distribution across all 134 publications.

A central challenge in assessing purportedly sustainable pavement systems lies in the substantiation of their environmental claims. This review, therefore, makes a foundational distinction between two categories of evidence: first, quantified environmental impacts derived from formal LCA methodologies, which provide systematic inventories of indicators such as global warming potential, cumulative energy demand, and abiotic resource depletion; and second, qualitative assertions of environmental benefit, often based solely on the recycled origin of constituent materials or the diversion of waste from landfill. Drawing this distinction is not merely taxonomic but essential for advancing the field from well-intentioned conceptual frameworks toward rigorously verified, performance-based engineering specifications.

While the performance and material composition of SMA have been extensively reviewed, a critical synthesis integrating advanced material science with contemporary sustainability paradigms remains absent. This review addresses that gap by constructing a unified analytical framework to systematically evaluate a broad range of modern additives from nanomaterials and recycled polymers to bio-based modifiers and industrial byproducts. Within this framework, we critically assess the efficacy of these materials in enhancing key engineering properties, including rutting resistance, fatigue life, and moisture durability, while concurrently evaluating their environmental footprints. Beyond synthesis, the analysis offers a critical comparative appraisal of additive benefits and limitations, supported by consolidated data, and identifies persistent research gaps, particularly concerning the long-term performance of sustainable SMA mixtures. Finally, we delineate prioritized future research trajectories aimed at optimizing SMA for dual objectives: exceptional structural resilience and reduced ecological impact. By explicitly bridging technical innovation with lifecycle and circular economy principles, this work serves as a holistic reference designed to guide the development of next-generation, high-performance, and environmentally responsible pavement systems.

## 2. Methodology

**Figure 5:**
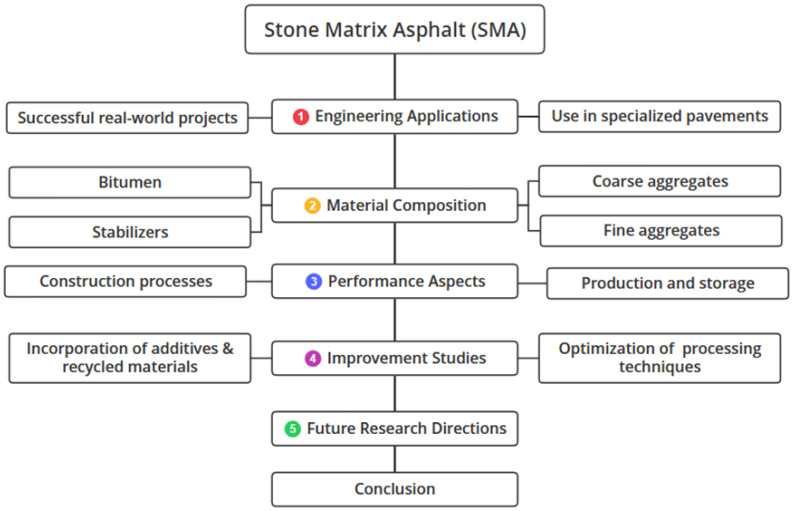
Methodology of the review paper.

This review systematically examines SMA across five areas: engineering applications in specialized pavements, material composition including aggregates and stabilizers, performance characteristics from production to durability, recent improvements using recycled materials and climate adaptations, and future research directions focusing on cost-effectiveness, nanotechnology, and sustainable development as illustrated in Figure 5. The analysis highlights SMA’s advantages while addressing current limitations and innovation opportunities.

## 3. Stone Matrix Asphalt (SMA): A Durable Solution for High-Traffic Areas, Bridges, and Airport Pavements

SMA is widely used in high-traffic areas due to its exceptional performance characteristics. Its ability to endure heavy loads and resist wear and tear makes it ideal for highways, bridges, and airport pavements. For example, the Virginia Department of Transportation has successfully implemented SMA on over 25% of its interstate network, demonstrating its durability and cost-effectiveness compared to traditional dense-graded asphalt [1]. Similarly, SMA has been extensively adopted on the German Autobahn, where it effectively handles heavy traffic and harsh environmental conditions [2].

In airport settings, SMA’s coarse aggregate structure provides the necessary load-bearing capacity for runways. The U.S. Air Force has constructed SMA runways in Germany and Italy, showcasing its effectiveness in airfield pavements [3]. On bridges, SMA is a popular choice due to its ability to handle high temperatures and resist deformation. When combined with Guss-mastic asphalt (GMA), it enhances structural strength and provides a smooth ride, even under heavy traffic. SMA also acts as a water-resistant barrier, preventing damage. Innovations like the SMA and Mastic Asphalt (MA) blend simplify application, making it a great fit for bridges exposed to challenging conditions over water [4,5].

The demand for specialized pavements in these applications stems from the need for enhanced durability, resistance to heavy loads, and improved skid resistance. SMA addresses these requirements through its unique composition, which includes a high bitumen content and a coarse aggregate skeleton. This results in reduced maintenance costs, extended service life, and a cost-effective solution for high-stress environments [6]. Real-world examples, such as the German Autobahn, Virginia highways, and U.S. Air Force runways, underscore SMA’s versatility, long-term performance, and suitability for demanding applications.

## 4. Composition of SMA: Key Components and Their Roles

**Figure 6:**
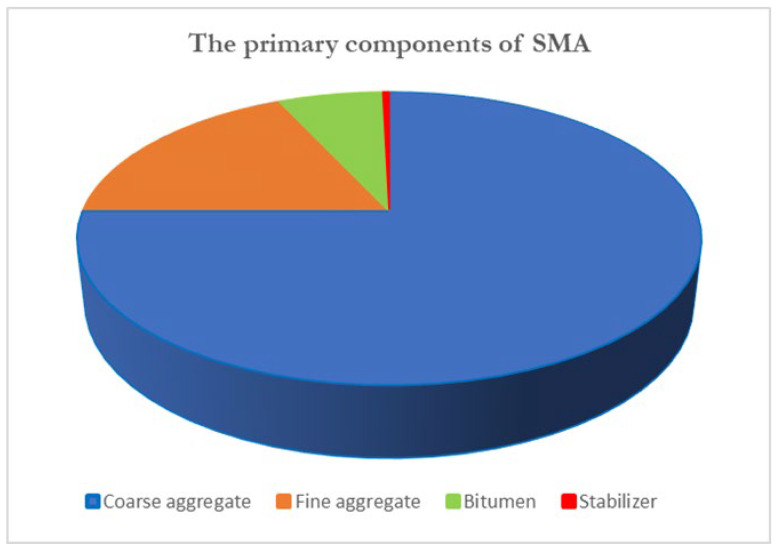
The primary components of SMA.

SMA is a highly durable paving material designed for heavy traffic, relying on a precise combination of key components to achieve its performance. As Figure 6 illustrates, the main components in SMA are coarse aggregates, fine aggregates, bitumen, and stabilizers.

1. Coarse Aggregates: Coarse aggregates constitute 70–80% of the SMA mix, forming the primary skeleton. The stone-on-stone contact between these aggregates provides structural integrity, load-bearing capacity, and resistance to rutting [7,8].
2. Fine Aggregates: Fine aggregates fill the voids between coarse aggregates, contributing to stability and workability. They also form a mastic with the bitumen, which coats the aggregates and enhances the overall strength of the mixture.
3. Bitumen: Bitumen, making up 6–7% of the mix, acts as the binder that holds the aggregates together. It provides flexibility and resistance to cracking under environmental and traffic stresses [9].
4. Stabilizers: Stabilizers, such as cellulose or polymer fibers, are added in small quantities (0.3–0.5% of the mix) to prevent bitumen drain-down during transport and placement. Mechanistically, these fibers create a three-dimensional network within the bitumen mastic. This network increases the viscosity and yield stress of the mastic at high production temperatures, effectively entrapping the binder and preventing its gravitational separation from the coarse aggregate skeleton. These fibers ensure uniform distribution of the binder and maintain the mix’s stability and durability [10].

Together, these components create a robust, rut-resistant pavement capable of withstanding demanding conditions [11].

The structural performance of SMA is fundamentally governed by its aggregate skeleton, a load-bearing network established through a deliberately gap-graded composition. This optimized gradation promotes a dense, stone-on-stone contact among coarse aggregates, creating the primary mechanical matrix responsible for resisting permanent deformation. Consequently, the incorporation of sustainable or alternative materials—including fine RAP, industrial by-products, or non-conventional fillers—necessitates a dual assessment. While their compatibility with the binder system is essential, their more critical evaluation lies in their potential to disturb the volumetric balance and interparticle contact of the coarse aggregate skeleton. A successful, sustainable mix design must strategically confine these materials to the mastic phase, ensuring they act as fillers without compromising the critical interlock of the primary load-bearing structure. Thus, gradation optimization remains an indispensable prerequisite in sustainable SMA development, a step that safeguards the mixture’s defining mechanical principle while integrating environmental advancements.

## 5. Steps Involved in Producing and Constructing SMA Pavements

The production of SMA involves high-temperature mixing, similar to other hot mix asphalts. However, the use of warm mix additives like Sasobit, Rheofalt, and Zycotherm can lower mixing temperatures, reducing energy consumption and emissions [12]. To prevent binder drainage during production and storage, fibers such as areca, coconut, or cellulose are added, with an optimal fiber content of around 0.3% by weight [13]. Careful handling is required during storage and transportation to mitigate drainage issues, which can also be addressed using rubber-modified binders as an alternative to fibers [14,15].

The laboratory design of SMA, which employs the Superpave Gyratory Compactor for volumetric verification, yields a mortar-rich, gap-graded mixture with a stable stone skeleton. Although its inherent stiffness presents construction challenges, these are often mitigated by warm mix asphalt technologies to enhance workability. This engineered composition provides superior in-service performance, characterized by high resistance to rutting and fatigue cracking. Furthermore, the resulting surface macrotexture contributes to improved skid resistance and noise attenuation. The system’s durability and functionality are ultimately secured through the interlocking aggregate structure and the stabilizing role of fibers or additives [16].

Overall, SMA production and storage require careful handling to prevent binder drainage, construction demands specialized equipment to manage its stiffness, and its road performance is significantly enhanced by additives and fibers, making it a durable and high-performing paving solution.

## 6. Additives and Technologies

**Figure 7:**
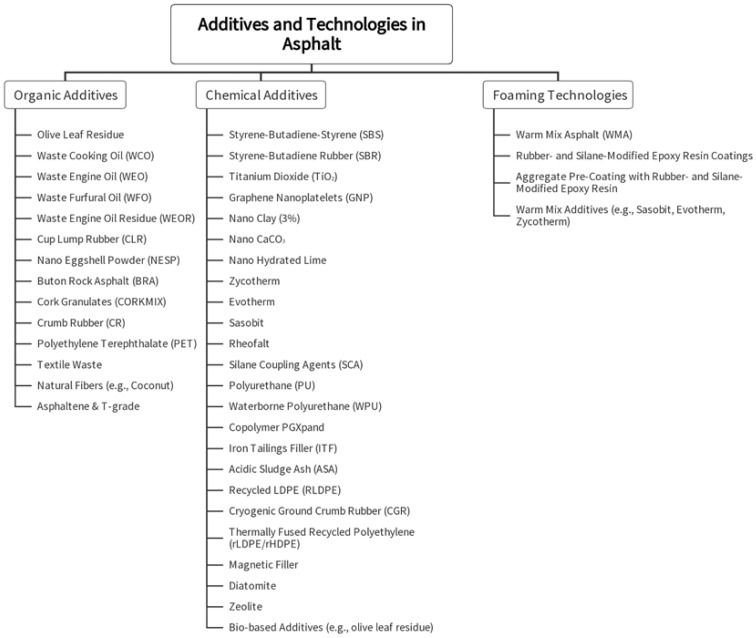
Categories of Additives and Technologies for Enhanced Asphalt Performance.

Asphalt’s performance, sustainability, and durability are enhanced through the use of various additives and technologies. As illustrated in Figure 7, these innovations fall into three key categories: Organic Additives, Chemical Additives, and Foaming Technologies.

The contemporary investigation of asphalt additives and production technologies delineates a definitive evolution, oriented toward the dual imperatives of superior engineering performance and enhanced environmental sustainability. Analysis of the current research landscape identifies three principal vectors of innovation: (1) Organic Additives, derived from industrial, agricultural, and municipal waste streams (e.g., crumb rubber, waste oils); (2) Chemical Additives, encompassing engineered polymers, nanomaterials, and performance-modifying agents for precise control of material properties; and (3) Foaming Technologies, which reduce energy consumption through lower production temperatures and facilitate novel aggregate coating mechanisms. This trajectory is fundamentally characterized by a pronounced alignment with circular economy principles, wherein recycled and bio-based constituents are strategically utilized to mitigate ecological impact while maintaining, and in some cases enhancing, critical mechanical parameters. Notwithstanding these advances, persistent research gaps constrain the field’s progression. Paramount among these is a scarcity of long-term, in situ performance data for novel materials; a lack of standardized characterization protocols for heterogeneous, waste-derived modifiers; the need for rigorous, system-level lifecycle assessments to substantiate purported environmental benefits; and a limited understanding of the compatibilities and potential synergies arising from the blending of multiple additives. Addressing these deficiencies is a critical prerequisite for the maturation and codification of these promising laboratory innovations into reliable, standard practice within pavement engineering.

### 6.1. Advanced Materials for Improved Asphalt Rutting Performance

Asphalt mixtures are widely used in road construction due to their durability, though they have both strengths and limitations. Modified blends, such as those with styrene-butadiene-styrene (SBS) and crumb rubber (CR), improve elasticity, stiffness, and rutting resistance under high temperatures and heavy traffic [17,18,19]. High-performance synthetic fibers reduce permanent deformation by 40–50% in wheel-tracking tests. Notably, a 0.3% dosage of natural coconut fiber matches the efficacy of cellulose in controlling binder drainage, preserving the critical retention limit for mixture integrity [20]. Alternative fillers, including dry flue gas ash and nano-hydrated lime, boost SMA performance by improving rheological properties, stiffness, and moisture resistance [21,22]. Basalt aggregates and nano-clay (3%) further enhance rutting resistance [23], while polymer-modified asphalt (PMA) with SBS/SBR (optimal at 4.5% SBR) improves elasticity [24,25]. Nano-fibrous carbon (NFC) enhances high-temperature and aging resistance but requires careful dosing [26], and recycled polyethylene (rLDPE/rHDPE) creates long-lasting super-hydrophobic surfaces [27]. Acidic sludge ash (ASA) improves stability but may reduce low-temperature performance [28], whereas iron tailings (50% substitution) optimize SMA rutting resistance [29]. Buton rock asphalt (BRA) with basalt fiber (BF) further improves rutting and low-temperature performance [30]. Cork granules (CORKMIX) improve rut resistance but reduce fatigue life, while crumb rubber (RUBMIX) enhances stiffness and fatigue resistance [31].

### 6.2. Innovative Materials for Improved Asphalt Fatigue Performance

Waste tire textile fibers (WTTF) and end-of-life tire fibers significantly enhance fatigue performance, serving as sustainable alternatives that also improve binder stability, cracking resistance, and environmental sustainability [32]. Research on SMA has also focused on enhancing fatigue resistance, skid resistance, and noise reduction, with recycled polymeric fibers from tires significantly extending pavement lifespan [33,34]. Sustainable fillers like geothermal waste and sugarcane bagasse ash (SBA) improve SMA stability, stiffness, and moisture resistance while reducing costs [35]. Polyethylene foam (T92) enhances fatigue resistance by absorbing energy under cyclic loads [36], while grid-structured aggregates improve adhesion over groove-structured ones [37]. Large-stone asphalt mixtures (LSAM) exhibit superior fatigue resistance, influenced by loading conditions [38], and natural rock asphalt enhances cement toughness under extreme conditions [39].

Modifiers such as graphene nanoplatelets (GNP) and warm-mix agents improve cracking resistance, though GNP lacks moisture resistance [40], while optimized mixing reduces segregation [41]. EA-10 asphalt performs well in harsh environments due to its low-temperature crack resistance [42], and fractionalized PET enhances SMA’s resilient modulus, tensile strength, and fatigue life [43]. Polyester fibers (0.4%) increased the fracture energy in semi-circular bend tests by 35% and improved the Tensile Strength Ratio (TSR) to >85%, indicating superior cracking and moisture resistance in coastal environments [44], whereas glass fibers increase stiffness but reduce deformation resistance [45]. Cryogenically ground rubber (CGR) enhances low-temperature performance but raises viscosity concerns at higher contents [46], and recycled LDPE (4–8%) improves high-temperature performance but may reduce moisture resistance and fatigue life at higher doses [47].

Additionally, polymer-modified binders and organic additives like imidazolines enhance SMA’s viscoelastic properties, improving low-temperature cracking resistance and reducing maintenance costs [48]. These innovations collectively contribute to more durable and sustainable asphalt pavements.

### 6.3. Innovative Nano-Additives Boost SMA Performance and Sustainability

**Figure 8:**
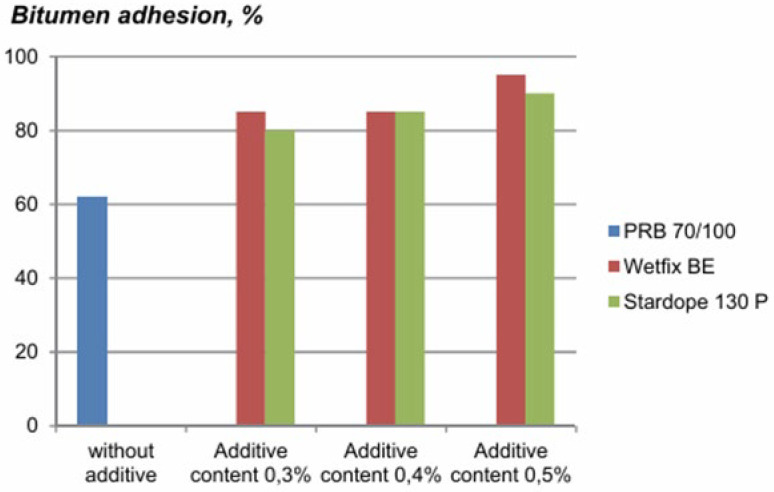
Effect of Additive Content on Bitumen Adhesion: A Comparative Analysis of PRB 70/100, Wetfix BE, and Stardope 130 P [49].

Additives and alternative binders play a crucial role in enhancing the performance, durability, and sustainability of asphalt pavements. As shown in Figure 8, adhesive additives improve adhesion and compressive strength across varying temperatures [49].

**Figure 9:**
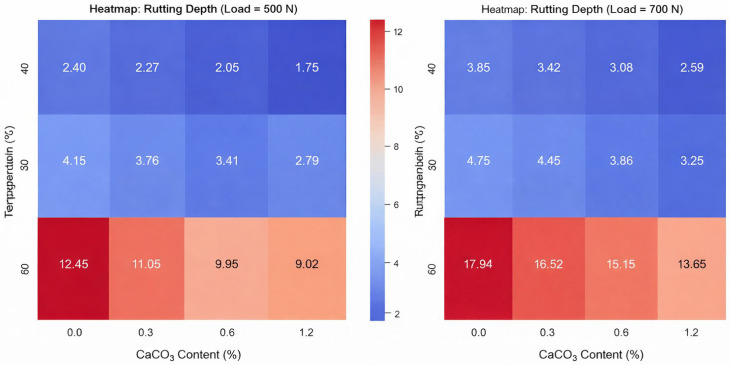
Visualizing the Impact of CaCO_3_ on Pavement Rutting Depth Across Temperature Gradients and Loads [63].

Bio-based additives, like olive leaf residue, serve as antioxidants and rheological modifiers. However, their efficacy varies depending on the type of base bitumen used [50,51]. Polymers like SBS and HDPE, along with nanomaterials such as graphene and diatomite, enhance ductility, thermal stability, and rutting resistance, with graphene notably improving Marshall stability [52,53,54,55]. Thermosetting polyurethane (TPUA) enhances high-temperature stability and flexibility, with 30 wt% PU content achieving optimal phase transition and surface free energy [56]. Titanium dioxide (TiO_2_) boosts tensile strength and moisture resistance, though it increases stiffness, with 2% TiO_2_ offering a balanced performance [57,58]. Magnetite fillers improve fracture toughness, fatigue life, and self-healing properties, making them ideal for sustainable pavements [59,60]. Nanomaterials such as nano Zinc Oxide (ZnO), nano Reduced Graphene Oxide (RGO), Zycotherm, and Evonik have been found to enhance moisture resistance in SMA mixtures. Among these, ZnO demonstrated superior effectiveness and cost-efficiency compared to RGO, while Zycotherm reduced moisture-induced strength loss by 15% more than Evonik in modified Lottman tests, demonstrating superior effectiveness in mitigating moisture damage [61]. Incorporating 0.9% nano CaCO_3_ improved fatigue life by 40–60%, reduced rutting depth by 30%, and increased moisture resistance by 25% compared to the control mix [62]. Similarly, another study highlighted the benefits of nano-hydrated lime and nano-CaCO_3_ in enhancing moisture resistance. As Figure 9 demonstrates, nano-hydrated lime proved to be the most effective additive tested [63].

Nanotechnology enhances SMA’s moisture resistance and mechanical properties through nano-additives like nano-CuO, nano-Al_2_O_3_, CNT, and HDPE, which improve binder performance and pavement durability [64,65,66] Recycled materials, such as rPVC, boost stability and thermal resistance, while ceramic waste provides thermal insulation, albeit with slightly reduced stability [67,68]. Self-healing asphalt mechanisms aim to extend pavement lifespan by repairing microcracks, reducing maintenance and environmental impact [69]. Additives like NESP improve hardness and aging resistance, and WPU-modified epoxy asphalt lowers energy consumption [70,71]. The copolymer PGXpand outperforms SBS modifiers with lower dosages and temperatures [72], while organic additives like Asphaltene and T-grade enhance viscosity, durability, and temperature stability [73]. These advancements collectively contribute to more sustainable and long-lasting pavements.

**Figure 10:**
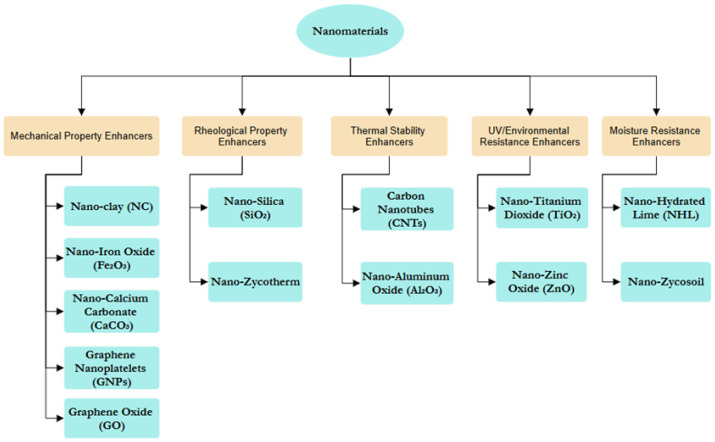
Additives Used in Asphalt Modification.

Figure 10 categorizes nanomaterials into five groups based on their enhanced properties: mechanical strength, rheological behavior, thermal stability, UV/environmental resistance, and moisture resistance. This classification shows how these materials can be customized to improve performance in areas like durability, flow control, heat resistance, and protection against environmental factors.

The targeted incorporation of nanoscale additives, such as nano-silica, carbon nanotubes, and functionalized clays, offers a potent strategy for enhancing the mechanical, rheological, and environmental resilience of asphalt binders, particularly in specialized mixtures like Stone Mastic Asphalt. This approach enables precise material engineering, moving beyond conventional modification towards multifunctional solutions that improve fatigue life, rutting resistance, and moisture susceptibility. However, the path to standardization requires bridging critical gaps, most notably through long-term field validation, comprehensive lifecycle assessments, and the development of robust protocols for nanomaterial dispersion and hybrid system compatibility.

### 6.4. Advanced Techniques in Sustainable Asphalt Mixtures: Enhancing Performance and Environmental Benefits

The addition of graphene nano-platelets (GNPs) or borosilicate glass significantly improves noise reduction and skid resistance, providing dual benefits for acoustics and safety [74,75]. Optimizing SMA with smaller nominal maximum aggregate sizes (NMAS) (5.6 mm or 8.0 mm) enhances crack resistance, reduces tire-pavement noise, and improves skid resistance and bond strength [76]. Field studies demonstrate that rubberized SMA and waste-plastic mixtures further enhance rutting and fatigue resistance, while open-graded friction courses (OGFC) improve noise and friction but require adjusted binder content for durability [77]. Double-layer porous asphalt (DLPA) offers superior noise control through optimized porosity and aggregate sizing [78].

Sustainable solutions like RAP in SMA mixtures improve moisture resistance and rutting performance while reducing environmental impact [79]. WMA technologies, such as Sasobit and Evotherm, lower production temperatures (20–40 °C reduction) and emissions without compromising performance [80]. Combining WMA with RAP enhances workability and durability [81], while bio-based recycling agents mitigate thermal cracking in cold climates [82,83]. Micro-surfacing with RAP boosts skid resistance and reduces emissions [84], and advanced modifications like rubber- and silane-modified epoxy resins in WMA further cut production temperatures (120–130 °C) while improving moisture resistance [85,86]. Other innovations include Portland cement (PC) in WMA and cup lump rubber (CLR) in cold mix asphalt (CMA) for better tensile strength [87], alongside surface activation techniques to enhance crumb rubber-asphalt compatibility [88].

LCA studies demonstrate that reclaimed asphalt pavement (RAP) in SMA mixtures (up to 30%) can improve performance while quantitatively reducing environmental impact, with typical savings of 15–25% in embodied carbon and energy [89], but issues like moisture in RAP need to be addressed [90]. Researchers have found that waste engine oil residue (WEOR) and CR allow for complete waste recycling, especially when enhanced by aqua-thermal curing and devulcanization methods, which improve their performance [91,92,93]. Additionally, the eco-friendly catalyst NA-NHSO_3_H, derived from natural asphalt, efficiently synthesizes heterocyclic compounds while adhering to green chemistry principles [94].

Based on previous studies, Table 1 summarizes the additives used in bitumen and asphalt mixtures, including their respective roles and effects. The table offers a detailed overview of how these additives influence performance, durability, and sustainability in asphalt applications.

Drawing from prior research, Table 2 presents a comparative analysis of additives and alternative binders, highlighting their benefits, limitations, and relevant literature sources.

### 6.5. Advanced Materials for Enhanced Asphalt Performance

**Figure 11:**
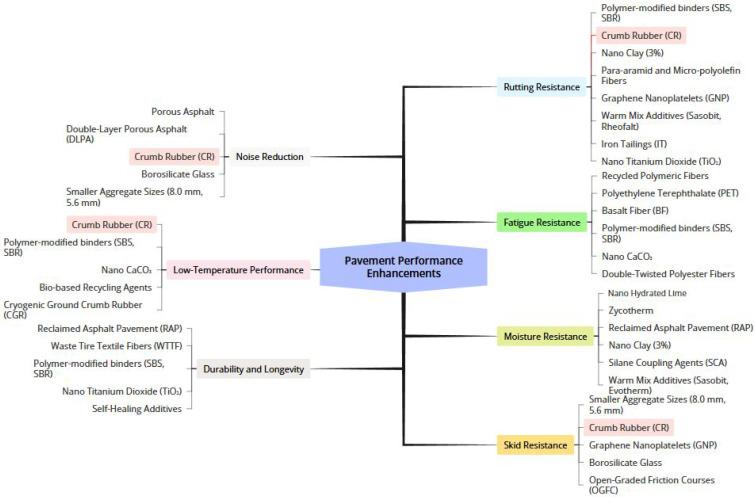
Essential Materials for Improved Pavement Performance.

Figure 11 organizes pavement improvements into six critical areas: rutting resistance, noise reduction, fatigue resistance, low-temperature performance, moisture resistance, and skid resistance. CR and polymer-modified binders (SBS/SBR) emerge as versatile solutions across multiple categories. Advanced nanomaterials like nano-clay and titanium dioxide show promise for targeted enhancements, while recycled materials contribute to sustainable performance gains. The clear categorization helps engineers and researchers quickly identify optimal solutions for specific pavement challenges.

### 6.6. Rubberized Asphalt: Sustainable Solutions for Durable Pavements

CR, used since the 1840s, enhances asphalt durability and sustainability [95]. Studies show that rubberized mixtures improve fracture resistance at low temperatures, reduce drain-down, and lower production costs [100]. Blending CR with modifiers like SBS or waste engine oil (WEO) boosts stability and flexibility, though long-term aging can increase brittleness [96,97,101]. Waste materials, such as textile fibers and pyrolyzed tire rubber, further enhance mechanical properties while supporting circular economy goals [102,103,104].

Innovative techniques, including chemically activated rubber (CARA) and functionalized graphene oxide (MT-GO), improve viscoelasticity, thermal resistance, and emission reduction [105,106]. Hybrid modifiers like SBS and recycled polyethylene optimize performance, while rejuvenators (waste furfural oil, waste cooking oil) restore aged asphalt [107,108,109,110,111]. Environmental assessments confirm that rubberized mixtures reduce resource consumption and emissions, though leaching risks require management [112,113].

Recent research demonstrates significant progress in crumb rubber-modified asphalt (CRMA), showcasing its potential for specialized applications such as railway tracks and urban pavements, where it provides noise reduction, freeze–thaw resistance, and crack mitigation [114,115]. Key advancements include optimizing organoclay surfactant loading at 85% of the cation exchange capacity (CEC) to enhance compatibility and aging resistance, as well as combining SBS copolymers with crumb rubber to improve thermal stability and mechanical performance [114,115]. Additionally, the incorporation of dynamic disulfide bonds has been shown to enhance self-healing properties, while controlled depolymerization of crumb rubber improves blend homogeneity [116,117]. Furthermore, biological desulfurization techniques have proven effective in increasing storage stability by reducing sulfur content [118,119]. These innovations collectively enhance the durability, sustainability, and cost-effectiveness of rubberized asphalt, though further research is needed to address remaining challenges in large-scale implementation. The main features of the studies are summarized in a comparison table (Table 3).

**Figure 12:**
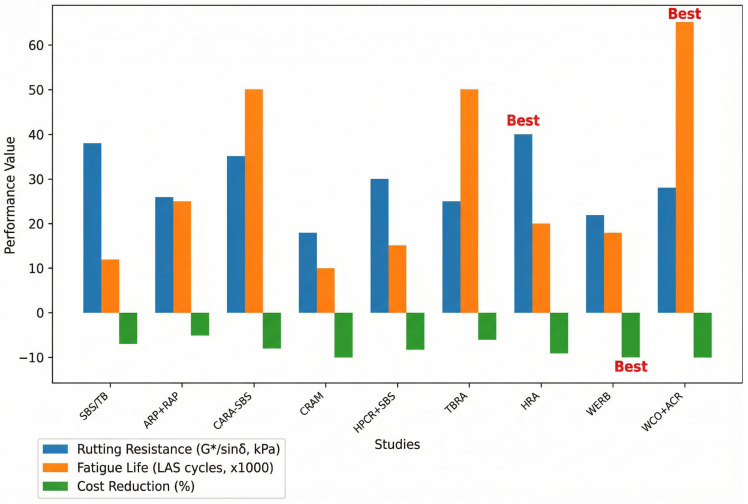
Comparison of Asphalt Modification Methods: Performance Metrics (Rutting Resistance, Fatigue Life, and Cost Reduction).

Studies show optimal modifier contents (3% CR, 40% TBRA, 18% ACR) beyond which performance stagnates or declines. Mixing typically occurs at 160–220 °C, with higher temperatures for CR-rich blends. SBS/rubber modifiers enhance thermal, rheological, and mechanical properties, while waste materials (CR, WCO, RAP) improve sustainability without compromising performance. The performance characteristics of various asphalt modification techniques, including rutting resistance, fatigue life, and cost reduction, are quantitatively compared in Figure 12.

Figure 12 presents a multi-criteria comparative analysis of nine asphalt modification strategies, quantifying their performance across three pivotal parameters: rutting resistance, fatigue life, and cost reduction potential. The results reveal distinct performance-cost trade-offs among the technologies. Notably, the highly rejuvenated asphalt (HRA) blend yields the greatest resistance to permanent deformation, whereas formulations incorporating waste cooking oil and acrylate copolymer (WCO + ACR) exhibit the most extended fatigue life. The waste engine oil-based rejuvenating binder (WERB) strategy demonstrates the highest potential for economic savings. This triaxial evaluation provides a foundational framework for a performance-driven, economically informed selection process in advanced pavement design, enabling engineers to optimize material choice against specific project constraints and objectives.

A critical perspective on additive selection must move beyond cataloguing performance enhancements to confront the central trade-offs of cost, scalability, and material trade-offs. While advanced materials like graphene nanoplatelets offer exceptional mechanical gains, their prohibitive cost and dispersion challenges starkly contrast with the favorable economics and waste valorization of CR or RAP, albeit with potential workability sacrifices. The literature further reveals a troubling context-dependency; findings for modifiers like nano-clay or recycled plastics are often contradictory, hinging on base binder specifics and compounding the challenge of universal application. Crucially, a gulf persists between commercially mature technologies (e.g., polymer modification, warm-mix additives) and promising yet low-TRL innovations like self-healing systems or engineered nanomaterials. The latter lack validated field durability, scalable production, and cost-effective integration pathways. Consequently, advancing sustainable SMA necessitates a developmental pathway that prioritizes holistic life-cycle assessments, standardized long-term field trials, and explicit research into industrial-scale integration, ensuring laboratory promise translates into practical, viable innovation.

## 7. Challenges and Future Directions for Sustainable SMA with Recycled Materials

Significant research gaps remain in understanding the long-term performance of SMA incorporating recycled aggregates under various environmental conditions, particularly regarding the effectiveness of fiber reinforcement in addressing durability concerns [123]. Future investigations should prioritize optimizing SMA mixtures using locally available recycled materials and industrial byproducts to achieve sustainable pavement solutions without compromising performance [124,125].

Recent advancements in asphalt technology have focused on improving material performance while alternative waste materials like coal slag show potential for improving Warm Mix Asphalt properties [126]. Innovative composite modifiers combining thermoplastic polyurethane with waste rubber have demonstrated improved elastic recovery and aging characteristics [127]. Researchers have also developed intelligent classification systems to address quality variability in high-RAP mixtures [128], and explored nano-modification techniques to improve crumb rubber compatibility [129].

Emerging sustainable solutions include self-healing SMA mixtures incorporating shape-memory alloys and fly ash [130], advanced interfacial agents for recycled asphalt [131], and novel pelletized additives combining waste materials with rejuvenators for high-RAP mixes [132]. Environmental protection measures such as VOC-reducing additives (zeolite and carbon black) have shown promising laboratory results [133]. However, these innovations require further validation through long-term performance monitoring and field-scale implementation to ensure practical applicability.

A critical barrier to the adoption of sustainable asphalt technologies is the unresolved disparity between optimized laboratory formulations and proven, long-term field performance. Research robustly demonstrates that mixtures incorporating recycled components—such as RAP or CR—and enhanced with polymers or nanomaterials exhibit superior mechanical properties under controlled conditions. However, their practical viability remains uncertain due to a lack of conclusive validation for essential durability metrics. These include prolonged aging characteristics, moisture damage resistance amidst seasonal freeze–thaw cycles, and fatigue life under complex, variable traffic loading. Crucially, conventional accelerated laboratory protocols cannot adequately replicate the synergistic and often extreme interactions of environmental and mechanical stressors encountered in service. Consequently, transitioning these advanced mixtures from experimental promise to codified practice necessitates the systematic acquisition of long-term performance data. This can only be achieved through well-monitored, instrumented pavement sections that provide real-world evidence under actual climatic and traffic conditions.

## 8. Conclusions

SMA has firmly established itself as a premier paving solution for high-stress infrastructure applications due to its exceptional durability, resistance to deformation, and long-term performance under demanding conditions. Extensive research and real-world implementations—from the German Autobahn to U.S. airport runways—have demonstrated SMA’s ability to withstand heavy traffic loads, extreme weather, and prolonged wear while maintaining structural integrity. The material’s unique composition, characterized by a coarse aggregate skeleton stabilized with fibers and high bitumen content, provides superior mechanical properties that outperform conventional asphalt mixes in rutting resistance, fatigue life, and skid resistance.

Recent advancements have further enhanced SMA’s sustainability profile through the incorporation of recycled materials such as RAP, crumb rubber from end-of-life tires, and industrial byproducts like waste textile fibers. These innovations not only reduce reliance on virgin resources but also address waste management challenges, contributing to a circular economy. Additionally, the adoption of WMA technologies has significantly lowered energy consumption and emissions during production, aligning SMA with global environmental objectives. Bio-based binders and rejuvenators have also emerged as promising alternatives, offering comparable performance while reducing the carbon footprint of pavement construction.

Despite these advancements, several challenges persist. Moisture sensitivity remains a critical concern, particularly in climates with freeze–thaw cycles, necessitating further research into moisture-resistant additives and modified mix designs. The high energy requirements for SMA production, though mitigated by WMA, still present opportunities for optimization, especially in regions with limited access to advanced manufacturing technologies. Moreover, while recycled materials show great promise, their long-term performance—particularly in terms of aging, binder compatibility, and load-bearing capacity—requires comprehensive field validation to ensure reliability over decades of service.

Future research directions should prioritize interdisciplinary approaches to address these gaps. Key areas include:

- Local Material Optimization: Developing SMA mixes tailored to regionally available aggregates and recycled materials to reduce transportation costs and environmental impact.
- Nanotechnology Integration: Exploring nano-additives (nano-clay, graphene) to enhance moisture resistance, self-healing properties, and mechanical performance.
- Eco-Friendly Innovations: Expanding the use of bio-based modifiers, low-emission production techniques, and LCA tools to quantify environmental benefits.
- Performance Modeling: Advanced predictive models to simulate long-term behavior under diverse climatic and traffic conditions, ensuring adaptability to future infrastructure demands.

In conclusion, while Stone Mastic Asphalt (SMA) exemplifies a synergistic balance of performance, economy, and sustainability, its maturation as a cornerstone of sustainable pavement engineering necessitates a critical and strategic framework. The literature converges on a pragmatic trajectory: the greatest gains in viable, sustainable SMA are likely achieved not through novel, high-cost additives, but via the optimized integration of established technologies. These include moderate reclaimed asphalt pavement (RAP) content (20–30%), warm-mix asphalt (WMA) techniques, and well-understood polymer modifiers. This pragmatism, however, must explicitly acknowledge inherent performance trade-offs. For instance, formulations optimized for superior rutting resistance may compromise low-temperature flexibility, while high recycled content can impair fatigue resistance without adequate rejuvenation. Consequently, future SMA design must be inherently performance-balanced and explicitly tailored to local climatic regimes, traffic spectra, and material availability.

Realizing the full potential of SMA for 21st-century infrastructure—from dense urban corridors to climate-resilient networks—demands collaborative, targeted innovation among researchers, engineers, and policymakers to elevate this adaptable material into a global benchmark for sustainable construction. It is crucial to note, however, that the promising laboratory results synthesized in this review highlight a significant knowledge gap. Critical factors including long-term durability, aging behavior, moisture susceptibility, and in-service field performance of modified and recycled SMA mixtures remain insufficiently examined. Many current conclusions are extrapolated from laboratory-scale data, which may not accurately replicate complex, long-term pavement behavior under real-world conditions. Therefore, future research must prioritize comprehensive field validations, long-term performance monitoring, and integrated laboratory-field studies. Bridging this gap is essential to translate advanced SMA mixtures from promising prototypes into reliably engineered, practical solutions.

**Figure 13:**
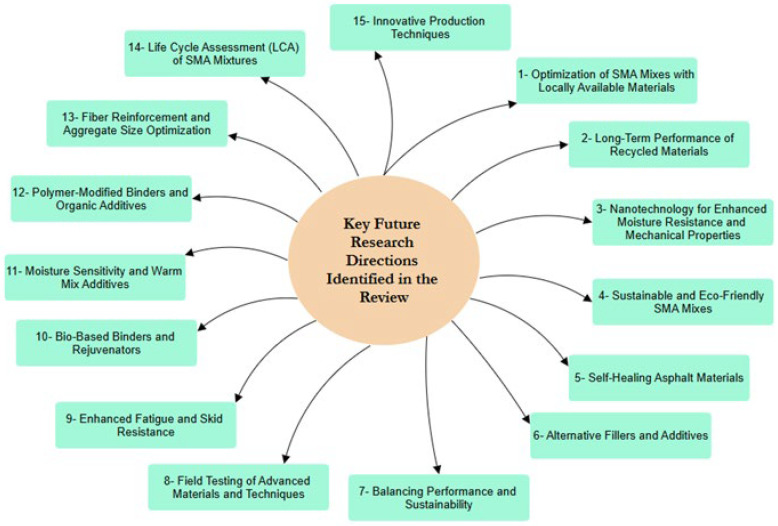
Summary of the future research directions.

Figure 13 delineates a strategic research agenda comprising 15 principal directions for SMA technology. These priorities coalesce around three core themes: the pursuit of sustainability through bio-based binders and recycled materials; the enhancement of mechanical performance, particularly in rutting and fatigue resistance, including self-healing capabilities; and the adoption of innovative techniques such as nanotechnology and fiber reinforcement. Collectively, they signal a field evolving toward eco-conscious materials science, with an emphasis on advanced functionalities and extended service life. Crucially, translating this promising laboratory research into routine practice necessitates a dedicated effort to close the pervasive gap between innovation and implementation. To this end, future work should systematically address the following interconnected challenges:

- Developing Realistic Aging Frameworks: There is a pressing need for standardized, multi-variable aging protocols that accurately simulate the long-term synergistic effects of environmental and mechanical stresses on modified and recycled SMA mixtures.
- Quantifying Novel Material Interactions: The integration of next-generation materials, including bio-rejuvenators and nano-modifiers, requires the establishment of clear mechanistic models and performance-based specification limits to guide their effective use.
- Implementing Holistic Design Methodologies: Optimization of SMA systems requires integrated digital tools that couple LCA with mechanistic-empirical design principles, enabling engineers to balance sustainability objectives with durability requirements in a unified framework.

Ultimately, the widespread adoption of advanced SMA mixtures will depend not solely on demonstrable performance gains, but on the parallel development of the pragmatic specifications, robust economic models, and practical design tools that empower infrastructure stakeholders to deploy them with confidence.

## Figures and Tables

**Table 1:** Summary of the additives used in Bitumen and Asphalt Mixture.

Additive	Added to Bitumen	Added to Asphalt Mixture	Role/Effect	References
**Polyethylene Foam (e.g., T92)**	Yes	No	Improves fatigue resistance and reduces deformation under cyclic loading.	[40]
**CR**	Yes	Yes	Enhances durability, elasticity, and sustainability; reduces drain-down.	[22,93]
**Graphene Nanoplatelets (GNP)**	Yes	Yes	Improves cracking resistance but lacks moisture resistance.	[44]
**Warm-Mix Agents (e.g., Sasobit)**	Yes	Yes	Reduces mixing and compaction temperatures, improving sustainability.	[13,34]
**Low-Density Polyethylene (LDPE)**	Yes	Yes	Improves stability and high-temperature performance.	[94]
**Waste Engine Oil (WEO)**	Yes	Yes	Enhances flexibility and reduces stiffness; improves workability.	[94]
**Basalt Fiber (BF)**	No	Yes	Enhances rutting and low-temperature performance.	[34]
**Polyethylene Terephthalate (PET)**	No	Yes	Improves resilient modulus, tensile strength, and fatigue life.	[47]
**Ceramic Waste**	No	Yes	Enhances thermal insulation but may reduce stability.	[72]
**Textile Waste**	No	Yes	Boosts mechanical properties and supports waste management.	[95]
**Nano Clay (3%)**	Yes	Yes	Enhances rutting resistance and oxidative aging resistance.	[12]
**SBS**	Yes	Yes	Improves rutting resistance, elasticity, and high-temperature performance.	[28,29]
**Styrene-Butadiene Rubber (SBR)**	Yes	Yes	Enhances rutting resistance and elasticity; optimal content at 4.5%.	[28,29]
**Nano Fibrous Carbon (NFC)**	Yes	Yes	Improves high-temperature performance and oxidative aging resistance.	[30]
**Acidic Sludge Ash (ASA)**	No	Yes	Enhances stability and moisture resistance but may reduce low-temperature performance.	[51]
**Recycled LDPE (RLDPE)**	Yes	Yes	Improves high-temperature performance but reduces moisture resistance at higher concentrations.	[41]
**Silane Coupling Agents (SCA)**	Yes	Yes	Improves adhesion, moisture resistance, and flexibility.	[42]
**Polyester Fibers (0.4%)**	No	Yes	Enhances cracking resistance, especially in coastal environments.	[48]
**Glass Fibers**	No	Yes	Increases stiffness and fatigue life but reduces resistance to permanent deformation.	[49]
**Cork Granulates (CORKMIX)**	No	Yes	Improves rut and wear resistance but reduces fatigue performance.	[35]
**Iron Tailings Filler (ITF)**	No	Yes	Reduces high-temperature performance and fatigue resistance unless modified with SCA.	[33]
**Cryogenic Ground Crumb Rubber (CGR)**	Yes	Yes	Improves low-temperature cracking resistance but increases viscosity.	[50]
**Thermally Fused Recycled Polyethylene (rLDPE/rHDPE)**	Yes	Yes	Transforms asphalt surfaces to super-hydrophobic, maintaining properties for two years.	[31]
**Magnetite Filler**	No	Yes	Improves microwave healing, fracture toughness, and self-healing properties.	[64]
**Titanium Dioxide (TiO_2_)**	Yes	Yes	Enhances rutting resistance and mechanical properties but may increase stiffness.	[62,63]
**Polyurethane (PU)**	Yes	Yes	Improves high-temperature stability, mechanical strength, and low-temperature flexibility.	[61]
**Bio-based Additives (e.g., olive leaf residue)**	Yes	Yes	Acts as antioxidants and rheological modifiers, improving mechanical properties.	[54,55]
**RAP**	No	Yes	Enhances sustainability by recycling materials; improves moisture resistance and rutting performance.	[28,89]
**Waste Tire Textile Fibers (WTTF)**	No	Yes	Stabilizes binder, improves cracking resistance, and supports waste management.	[36,37]
**Para-aramid and Micro-polyolefin Fibers**	No	Yes	Enhances resistance to permanent deformation and rutting.	[29]
**Natural Fibers (e.g., coconut)**	No	Yes	Prevents binder drain-down and enhances structural integrity.	[30]
**Geothermal Waste**	No	Yes	Improves stability and tensile strength while reducing costs.	[38]
**Dry Flue Gas Desulfurization Ash**	No	Yes	Enhances rheological properties and sustainability.	[25]
**Self-healing Additives**	Yes	Yes	Promotes microcrack healing, extending pavement lifespan.	[74]

**Table 2:** Summary of the various additives, highlighting their benefits and drawbacks.

Additive	Advantages	Disadvantages	References
**Adhesive Additives (Wetfix BE, Stardope 130 P)**	Improves adhesion and compressive strength across temperatures.	Effectiveness may vary with binder type.	[53]
**Olive Leaf Residue**	Acts as antioxidant and rheological modifier.	Effectiveness depends on base bitumen.	[54,55]
**Polymers (SBS, HDPE)**	Enhances ductility, thermal stability, and rutting resistance.	May reduce flexibility and homogeneity in mixtures.	[56,57,58,59,60]
**Graphene**	Significantly boosts Marshall stability and thermal stability.	High cost and potential dispersion challenges.	[56,57,58,59,60]
**Thermosetting Polyurethane (TPUA)**	Improves high-temperature stability and flexibility; optimal at 30 wt% PU.	Requires precise dosage for optimal performance.	[61]
**Titanium Dioxide (TiO_2_)**	Enhances tensile strength and moisture resistance.	Increases stiffness; optimal at 2% dosage.	[62,63]
**Magnetite Fillers**	Improves fracture toughness, fatigue life, and self-healing properties.	May increase material cost.	[64,65]
**Evonik & Zycotherm**	Reduces moisture susceptibility; Zycotherm outperforms Evonik in SMA.	Effectiveness varies with mixture type.	[66]
**Portland Cement (PC)**	Improves tensile strength, rutting resistance, and durability in WMA.	May increase stiffness and brittleness.	[86,87]
**Cup Lump Rubber (CLR)**	Enhances performance in cold mix asphalt (CMA).	Limited research on long-term durability.	[86,87]
**Nano Eggshell Powder (NESP)**	Improves hardness and aging resistance.	Requires further research on large-scale application.	[75]
**Waterborne Polyurethane (WPU)**	Reduces energy consumption in epoxy asphalt.	May have higher initial cost.	[76]
**Copolymer PGXpand**	Surpasses SBS modifiers; requires lower dosages and temperatures.	Limited availability and higher cost compared to traditional modifiers.	[77,78]
**Iron Tailings (IT)**	Improves rutting resistance in SMA; optimal at 50% substitution.	Potential variability in material quality.	[33]
**Asphaltene & T-grade**	Improves viscosity, durability, and lifespan; reduces temperature susceptibility.	May increase stiffness.	[79]
**Waste Cooking Oil (WCO)**	Cost-effective, eco-friendly alternative binder.	May require additional processing for optimal performance.	[96]
**Waste Engine Oil (WEO)**	Reduces reliance on fossil fuels; cost-effective.	Potential variability in quality and performance.	[96]
**Waste Furfural Oil (WFO)**	Restores aged asphalt; maintains viscoelastic properties.	Limited research on long-term performance.	[97]
**Waste Engine Oil Residue (WEOR)**	Potential for full waste recycling; improves performance with curing techniques.	Requires additional processing steps.	[89,90,91,98]
**CR**	Enhances elasticity and fracture resistance; promotes recycling.	May reduce mixture homogeneity and flexibility.	[89,90,91]
**Recycled PVC (rPVC)**	Enhances stability and thermal resistance; promotes sustainability.	Potential variability in material quality.	[73]
**Double-Twisted Polyester Fibers**	Increases peak load, fracture energy, and post-peak energy.	Minimal impact on DTP parameters; may reduce flexibility.	[99]
**NA-NHSO_3_H Catalyst**	Eco-friendly, reusable, and efficient for synthesizing heterocyclic compounds.	Requires specialized synthesis process.	[94]
**Nano CaCO_3_**	Improves fatigue life, rutting resistance, moisture resistance and better bonding.	Excessive use >0.9% reduces benefits and may increase strain, and mixing precision to ensure homogeneity.	[67]

**Table 3:** Comparison of Methodologies and Findings Across Studies.

Study	Base Material	Modifiers	Mixing Method	Key Findings	References
**SBS/TB Crumb Rubber**	70# asphalt	2–3% SBS, 10–15% TB crumb rubber	High shear mixing (4000 rpm), 180 °C, 1 h homogenization	Best performance: 3% SBS + 15% TB. Improved thermal stability, rutting resistance, and low-temperature flexibility.	[109]
**ARP with RAP**	Pen60/70 asphalt, 30% RAP	2% Activated Rubber Pellets (ARP)	Semi-wet process, 175 °C, 60 min curing	ARP enhances aged binder mobilization. Best for cracking resistance (75 IDEAL-CT index).	[120]
**CARA-SBS**	70# base asphalt	15% crumb rubber, 1% chemical additive, 2% SBS, 0.2% sulfur	200 °C, 6 h stirring, 180 °C for SBS/sulfur	Superior aging resistance due to crosslinked network. Minimal viscosity increases and best fatigue resistance.	[103]
**CRAM (Dry Process)**	70# asphalt	3–6% crumb rubber (2.36–4.75 mm)	Dry mixing at 180 °C, Marshall compaction	3% CR optimal: balances strength (45% Pmax reduction) and ductility (20% GF increase).	[106]
**HPCR with SBS/LDPE**	70# asphalt	20% HPCR (WCO-pretreated), 2–4% SBS, 0.55–0.6% sulfur	High-shear mixing (4500–5000 rpm), 185 °C	Best formulation: 20%HPCR + 4%SBS + 0.6%S. High ductility, rutting resistance (G*/sinδ), and low-temperature performance.	[121]
**TBRA (High CR Content)**	PG 64–16 asphalt	30–50% crumb rubber (40 mesh)	220 °C, 6-h shearing, overnight curing	40% CR optimal: uniform microstructure, best fatigue resistance (30× life vs. plain asphalt), moderate high-temperature performance.	[112]
**HRA (High Rubber Content)**	70# base asphalt	30–40% ARP, 2–3% SBS, 5–7% reinforcing agent, 0.4% stabilizer	180 °C, 4000 rpm shear, RSM optimization	HRA40: superior rheological performance, 60% lower fume emissions, and excellent thermal stability.	[122]
**WERB (WEOR + CR)**	Reclaimed bitumen (RB)	WEOR:CR ratios (3:1, 4:1), premixed at 180 °C	160–180 °C, 1500–4500 rpm mixing	4:1 WEOR:CR best: homogeneous microstructure, low HPI, and improved rheology (lower δ, higher G*).	[96]
**WCO + ACR**	Aged asphalt	7–10% WCO, 15–20% ACR (microwave-activated)	150–185 °C, 5000 rpm shearing	W10R18 (10% WCO + 18% ACR): optimal balance of high/low-temperature performance, fatigue resistance, and storage stability.	[95]

## Data Availability

No new data were created or analyzed in this study. Data sharing is not applicable to this article.

## Abbreviations

The following abbreviations are used in this manuscript:

SMA	Stone Matrix Asphalt
RAP	Reclaimed Asphalt Pavement
WTTF	Waste Tire Textile Fibers
WMA	Warm Mix Asphalt
GMA	Guss-mastic Asphalt
SBS	Styrene Butadiene Styrene
CR	Crumb Rubber
PMA	Polymer Modified Asphalt
NFC	Nano-fibrous Carbon
LSAM	Large-Stone Asphalt Mixtures
GNP	Graphene Nanoplatelets
CGR	Cryogenically Ground Rubber
TPUA	Thermosetting Polyurethane
OGFC	Open-Graded Friction Courses
DLPA	Double-Layer Porous Asphalt
LCA	Life Cycle Assessment
CRMA	Crumb Rubber-Modified Asphalt

## References

[1] Wu S., Wen H., Chaney S., Littleton K., Muench S. (2017). Evaluation of Long-Term Performance of Stone Matrix Asphalt in Washington State. J. Perform. Constr. Facil..

[2] Ai C., Qiu S., Xin C., Yang E., Qiu Y. (2016). Evaluation and optimisation of stone matrix asphalt (SMA-13) mix design using weight-matrix method. Road Mater. Pavement Des..

[3] Lin B., Wang Y.D., Liu J. (2025). Development of A balanced mix design approach for stone Matrix asphalt mixtures. Clean. Mater..

[4] Budziński B., Mieczkowski P. (2020). Application of Innovative SMA-MA Mixtures on Bridges. Appl. Sci..

[5] Sha A., Jiang W., Shan J., Wu W., Li Y., Zhang S. (2022). Pavement structure and materials design for sea-crossing bridges and tunnel: Case study of the Hong Kong–Zhuhai–Macau Bridge. J. Road Eng..

[6] Prabakaran E., Vijayakumar A., Kumar D.V., Lin F., Pastor D., Kesswani N., Patel A., Bordoloi S., Koley C. (2025). An Experimental Study of Stone Matrix Asphalt with Different Fillers. Artificial Intelligence in Internet of Things (IoT): Key Digital Trends.

[7] Golsefidi S.S., Sahaf S.A. (2023). Optimization of stone matrix asphalt mixture design based on fracture mechanics approach. Eng. Fract. Mech..

[8] James T., Prowell B. (2022). Evaluation of High Los Angeles Abrasion Loss Aggregate in Stone Matrix Asphalt. Transp. Res. Rec. J. Transp. Res. Board.

[9] Oda S., Fernandes J.L., Ildefonso J.S. (2012). Analysis of use of natural fibers and asphalt rubber binder in discontinuous asphalt mixtures. Constr. Build. Mater..

[10] Gedik A. (2023). Utilising cellulose fibre in stone mastic asphalt. J. Croat. Assoc. Civ. Eng..

[11] Ameri M., Mohammadi R., Vamegh M., Molayem M. (2017). Evaluation the effects of nanoclay on permanent deformation behavior of stone mastic asphalt mixtures. Constr. Build. Mater..

[12] Pasetto M., Baliello A., Giacomello G., Pasquini E. (2017). Sustainable solutions for road pavements: A multi-scale characterization of warm mix asphalts containing steel slags. J. Clean. Prod..

[13] Chegenizadeh A., Tokoni L., Nikraz H., Dadras E. (2021). Effect of ethylene-vinyl acetate (EVA) on stone mastic asphalt (SMA) behaviour. Constr. Build. Mater..

[14] Manosalvas-Paredes M., Gallego J., Saiz L., Bermejo J.M. (2016). Rubber modified binders as an alternative to cellulose fiber–SBS polymers in Stone Matrix Asphalt. Constr. Build. Mater..

[15] Chegenizadeh A., Aung M.-O., Nikraz H. (2021). Ethylene Propylene Diene Monomer (EPDM) Effect on Asphalt Performance. Buildings.

[16] Sarang G., Lekha B.M., Krishna G., Shankar A.U.R. (2016). Comparison of Stone Matrix Asphalt mixtures with polymer-modified bitumen and shredded waste plastics. Road Mater. Pavement Des..

[17] Shirini B., Imaninasab R. (2016). Performance evaluation of rubberized and SBS modified porous asphalt mixtures. Constr. Build. Mater..

[18] Zhang J., Huang W., Hao G., Yan C., Lv Q., Cai Q. (2021). Evaluation of open-grade friction course (OGFC) mixtures with high content SBS polymer modified asphalt. Constr. Build. Mater..

[19] Hafeez M., Ahmad N., Kamal M.A., Rafi J., Haq M.F.U., Jamal, Zaidi S.B.A., Nasir M.A. (2019). Experimental Investigation into the Structural and Functional Performance of Graphene Nano-Platelet (GNP)-Doped Asphalt. Appl. Sci..

[20] Saedı S. (2024). Evaluation of the effect of para-aramid and micro-polyolefin fibers on permanent displacement in stone mastic asphalt. J. Sustain. Constr. Mater. Technol..

[21] Li K., Zhou Z., Zhang Y., Ying R. (2024). Feasibility Analysis of Resource Application of Dry Flue Gas Desulfurization Ash in Asphalt Pavement Materials. Coatings.

[22] Das A.K., Singh D. (2021). Evaluation of fatigue performance of asphalt mastics composed of nano hydrated lime filler. Constr. Build. Mater..

[23] İskender E. (2013). Rutting evaluation of stone mastic asphalt for basalt and basalt–limestone aggregate combinations. Compos. Part B Eng..

[24] Diab A., Enieb M., Singh D. (2019). Influence of aging on properties of polymer-modified asphalt. Constr. Build. Mater..

[25] Vamegh M., Ameri M., Chavoshian Naeni S.F. (2020). Experimental investigation of effect of PP/SBR polymer blends on the moisture resistance and rutting performance of asphalt mixtures. Constr. Build. Mater..

[26] Wang Z., Ye F. (2020). Experimental investigation on aging characteristics of asphalt based on rheological properties. Constr. Build. Mater..

[27] Murugan K.P., Balaji M., Kar S.S., Swarnalatha S., Sekaran G. (2020). Nano fibrous carbon produced from chromium bearing tannery solid waste as the bitumen modifier. J. Environ. Manag..

[28] Dalhat M.A., Adesina A.Y. (2020). Utilization of micronized recycled polyethylene waste to improve the hydrophobicity of asphalt surfaces. Constr. Build. Mater..

[29] Li S., Zhang Z., Si C., Shi X., Cui Y., Bao B., Zhang Q. (2025). Evaluation of the rheological properties of asphalt mastic incorporating iron tailings filler as an alternative to limestone filler. J. Clean. Prod..

[30] Ma J., Cui Y., Xing Y., Chen X., Wu J. (2024). Optimization and pavement performance of buton-rock-asphalt modified asphalt mixture with basalt-fibre. Case Stud. Constr. Mater..

[31] Pereira S.M.S., Oliveira J.R.M., Freitas E.F., Machado P. (2013). Mechanical performance of asphalt mixtures produced with cork or rubber granulates as aggregate partial substitutes. Constr. Build. Mater..

[32] Valdés-Vidal G., Calabi-Floody A., Mignolet-Garrido C., Díaz-Montecinos C. (2023). Effect of a New Additive Based on Textile Fibres from End-of-Life Tyres (ELT) on the Mechanical Properties of Stone Mastic Asphalt. Polymers.

[33] Valdés-Vidal G., Calabi-Floody A., Mignolet-Garrido C., Bravo-Espinoza C. (2024). Enhancing Fatigue Resistance in Asphalt Mixtures with a Novel Additive Derived from Recycled Polymeric Fibers from End-of-Life Tyres (ELTs). Polymers.

[34] Guo P., Li C., Lu W., Lv S., Duan H., Lv H., Ding L. (2025). Road performance and noise reduction characteristics of dense graded dry-process rubberised asphalt mixture. Constr. Build. Mater..

[35] Akarsh P.K., Ganesh G.O., Marathe S., Rai R. (2022). Incorporation of Sugarcane Bagasse Ash to investigate the mechanical behavior of Stone Mastic Asphalt. Constr. Build. Mater..

[36] Abedraba-Abdalla M., Thom N., Garcia-Hernández A., Li L. (2024). Particle loss mitigation in asphalt by the addition of polyethylene foam. Constr. Build. Mater..

[37] Ge J., Yu H., Qian G., Dai W., Zhang C., Shi C., Zhou H., Nian T., Zhong Y. (2024). Enhancement mechanism of aggregate surface roughness structure on interfacial properties of asphalt mixtures. Constr. Build. Mater..

[38] Meng Y., Lu Y., Kong W., Chen J., Zhang C., Meng F. (2024). Study on the influence factors of fatigue properties of large-stone asphalt mixtures based on semi-circular bending tests. Constr. Build. Mater..

[39] Zhang C., Wang J., Cai J., Li K., Hu C., Mei K., Cheng X. (2024). The effect of evolution of rock asphalt subjected to high temperature and high pressure on the mechanical properties of oil well cement. Constr. Build. Mater..

[40] Sarkar M.T.A., Elseifi M.A., Hossain Z. (2024). Effects of warm-mix additives, anti stripping agent, and graphene nanoplatelet on the cracking resistance, moisture susceptibility, and cost effectiveness of stone mastic asphalt. Constr. Build. Mater..

[41] Sun C., Li P., Ma Y., Gou X., Ma Z. (2024). Experimental simulation evaluation and control measures of paving segregation for the stone matrix asphalt mixtures. Constr. Build. Mater..

[42] Ren H., Qian Z., Chen T., Cao H., Qian L., Zhang X. (2024). Fracture resistance of asphalt mixtures used for bridge deck pavement in high-altitude and cold regions. Constr. Build. Mater..

[43] Ben Zair M.M., Jakarni F.M., Muniandy R., Hassim S., Ansari A.H., Elahi Z. (2024). Investigation of the fractionalized polyethylene terephthalate (PET) on the properties of stone mastic asphalt (SMA) mixture as aggregate replacement. Case Stud. Constr. Mater..

[44] Hu Y., Wu J. (2024). Low-temperature crack resistance of stone matrix asphalt mixtures under chloride salt dry-wet cycles. Eng. Fract. Mech..

[45] Wu M., Wang W., Cai H., Liang J., Hua J., Dong H., Zhao L., Zhang Y. (2022). Effects of Fiber Type on the Mechanical Properties of the Open-Graded Friction Course Mixture. ACS Omega.

[46] Badughaish A., Li J., Amirkhanian S., Zhou Q., Xiao F. (2025). Impact of chemical pre-treatment on crumb rubber for coating property of rubberized asphalt. J. Clean. Prod..

[47] Shishehboran M., Ziari H., Habibnejad Korayem A., Hajiloo M. (2021). Environmental and mechanical impacts of waste incinerated acidic sludge ash as filler in hot mix asphalt. Case Stud. Constr. Mater..

[48] Mielczarek M., Fornalczyk S., Słowik M. (2024). Evaluation of Rheological Properties of Polymer-Modified Asphalt Binders and Mastics with Organic Additive—Imidazoline. Sustainability.

[49] Dvorkin L., Marchuk V., Kuzlo M. (2024). Influence of adhesive additives on the properties of bitumen and asphalt mixtures. Zastita Mater..

[50] Loise V., Abe A.A., Porto M., Muzzalupo I., Madeo L., Colella M.F., Rossi C.O., Caputo P. (2024). Plant Waste-Based Bioadditive as an Antioxidant Agent and Rheological Modifier of Bitumen. Materials.

[51] D’Addio G., Oreto C., Viscione N., Veropalumbo R. (2024). Influence of Bio-Additives on Recycled Asphalt Pavements. Materials.

[52] Guvalov (Kapanakchi) A., Mammadov A. (2024). Preparation of effective nanomodified polymer-bitumen adhesives for asphalt concrete. E3S Web Conf..

[53] Aslan M., İskender E., Aksoy A. (2024). Performance Investigation of Diatomite Modified Asphalt Mixtures for Different Diatomite Ratios and Grinding Sizes. Turk. J. Civ. Eng..

[54] Pacholak R., Gardziejczyk W., Wasilewska M., Gierasimiuk P., Woszuk A. Effect of zeolite addition to modified bitumen for application in wearing course. Proceedings of the 8th International Conference on Road and Rail Infrastructure.

[55] Nqabisa S., Khamlich S., Oliver G. (2024). Enhanced stability of a three-dimensional graphene nanosheets networks modified asphalt mixture. E3S Web Conf..

[56] Yang F., Gong H., Cong L., Shi J., Guo G., Mei Z. (2022). Investigating on polymerization process and interaction mechanism of thermosetting polyurethane modified asphalt. Constr. Build. Mater..

[57] Diniz M.I.L., Lucena A.E.d.F.L., Neto O.d.M.M., de Moraes T.M.R.P., de Sousa T.M., Silva I.M., Félix C.T.R. (2025). Titanium dioxide as a modifier in asphalt mixtures: Mechanical behavior under aging conditions. Constr. Build. Mater..

[58] Jin X., Guo N., You Z., Wang L., Wen Y., Tan Y. (2020). Rheological properties and micro-characteristics of polyurethane composite modified asphalt. Constr. Build. Mater..

[59] Ameri M., Sadeghiavaz M. (2025). Using magnetite filler to enhance the microwave healing of asphalt mixtures. Case Stud. Constr. Mater..

[60] Afshin A., Behnood A. (2025). Nanomaterials in asphalt pavements: A state-of-the-art review. Clean. Waste Syst..

[61] Lyu L., Li D., Chen Y., Tian Y., Pei J. (2021). Dynamic chemistry based self-healing of asphalt modified by diselenide-crosslinked polyurethane elastomer. Constr. Build. Mater..

[62] Yarahmadi A.M., Shafabakhsh G., Asakereh A. (2022). Laboratory investigation of the effect of nano Caco3 on rutting and fatigue of stone mastic asphalt mixtures. Constr. Build. Mater..

[63] Razavi S.-H., Kavussi A. (2020). The role of nanomaterials in reducing moisture damage of asphalt mixes. Constr. Build. Mater..

[64] Chen Z., Ge J., Song W., Tong X., Liu H., Yu X., Li J., Shi J., Xie L., Han C. (2024). 20.2% Efficiency Organic Photovoltaics Employing a π-Extension Quinoxaline-Based Acceptor with Ordered Arrangement. Adv. Mater..

[65] Praveenkumar T.R., Vijayalakshmi M.M., Meddah M.S. (2019). Strengths and durability performances of blended cement concrete with TiO_2_ nanoparticles and rice husk ash. Constr. Build. Mater..

[66] Li J., Tang F. (2023). Effects of two metal nanoparticles on performance properties of asphalt binder and stone matrix asphalt mixtures containing waste high density polyethelene. Constr. Build. Mater..

[67] Huang Q., Qian Z., Hu J., Zheng D. (2020). Evaluation of Stone Mastic Asphalt Containing Ceramic Waste Aggregate for Cooling Asphalt Pavement. Materials.

[68] Al-Shawabkeh A.F., Awwad M.T., Ikhries I.I., Abu-Hamatteh Z.S., Al-Najdawi N.A. (2025). Assessing recycled polyvinyl chloride reinforced modified asphalt mixtures for sustainable paving applications. J. Clean. Prod..

[69] Liu Z., Cavalli M., Kaestner A., Poulikakos L., Kringos N. (2024). Characterizing the Self-Healing Asphalt Materials: A Neutron Imaging Study. Adv. Eng. Mater..

[70] Zghair Chfat A.H., Yaacob H., Mohd Kamaruddin N.H., Al-Saffar Z.H., Putra Jaya R. (2024). Effects of nano eggshell powder as a sustainable bio-filler on the physical, rheological, and microstructure properties of bitumen. Results Eng..

[71] Zhou X., Wang Z., Guo H., Wang X., Chen W., Liu J., Zhang H., Wan C. (2024). Property improvement of epoxy emulsified asphalt modified by waterborne polyurethane in consideration of environmental benefits. Case Stud. Constr. Mater..

[72] Al-Saffar Z.H., Hasan H.G.M., Alamri M., Al-Attar A.A., Hamad A.J., Abdulmawjoud A.A., Mezaal M.R., Elmagarhe A. (2024). Assessing the effects of copolymer modifier addition on asphalt attributes: Towards achieving performance optimization. Constr. Build. Mater..

[73] Dong W., Ma F., Fu Z., Hou Y., Dai J., Zhao Z., Fang R. (2024). Investigating the properties of a novel organic composite warm mix additive on SBS modified asphalt binder. Constr. Build. Mater..

[74] Hamid A., Ahmad N., Zaidi B., Khalid R.A., Hafeez I., Hussain J., Khitab A., Kırgız M.S. (2023). GlasSphalt: A Borosilicate Based Sustainable Engineering Material for Asphalt Pavements. Sustainability.

[75] Li W., Han S., Huang Q. (2020). Performance of Noise Reduction and Skid Resistance of Durable Granular Ultra-Thin Layer Asphalt Pavement. Materials.

[76] Baek C., Kwon O., Lee J. (2024). Laboratory and Field Performance Evaluation of NMAS 9.5, 8.0, and 5.6 mm SMA Mixtures for Sustainable Pavement. Sustainability.

[77] Wang D., Schacht A., Leng Z., Leng C., Kollmann J., Oeser M. (2017). Effects of material composition on mechanical and acoustic performance of poroelastic road surface (PERS). Constr. Build. Mater..

[78] Liao G., Zha J., Lu X., Wu W., Zhang W., Wang H., Zhang Z., Liu X. (2024). Spectral noise reduction of double-layer porous asphalt: From laboratory to field. Constr. Build. Mater..

[79] Kranthi N., Raj K., Ramesh A. (2024). Development of sustainable pavement: An experimental study of stone mastic asphalt prepared with warm mix additives and reclaimed asphalt pavement. IOP Conf. Ser. Earth Environ. Sci..

[80] Li Q., Sun G., Lu Y., Meng Y., Luo S., Gao L. (2021). Effects of warm-mix asphalt technologies and modifiers on pavement performance of recycled asphalt binders. J. Clean. Prod..

[81] Ziari H., Orouei M., Divandari H., Yousefi A. (2021). Mechanical characterization of warm mix asphalt mixtures made with RAP and Para-fiber additive. Constr. Build. Mater..

[82] Ashimova S., Teltayev B., Oliviero Rossi C., Caputo P., Eskandarsefat S. (2021). Organic-based recycling agents for road paving applications in cold-climate regions. Int. J. Pavement Eng..

[83] Pouranian M.R., Shishehbor M. (2019). Sustainability Assessment of Green Asphalt Mixtures: A Review. Environments.

[84] Wang A., Shen S., Li X., Song B. (2019). Micro-surfacing mixtures with reclaimed asphalt pavement: Mix design and performance evaluation. Constr. Build. Mater..

[85] Jun Yoo P., Sik Eom B., Soo Park K., Hun Kim D. (2017). Aggregate pre-coating approach using rubber- and silane-coupled thermoset polymer and emulsion for warm-mix asphalt mixtures. Constr. Build. Mater..

[86] Viscione N., Veropalumbo R., Oreto C., Biancardo S.A., Abbondati F., Russo F. (2022). Additional procedures for characterizing the performance of recycled polymer modified asphalt mixtures. Measurement.

[87] Farahi B., Manjili M.H., Ghahremani M., Aghayan I., Faheem A., Sobolev K. (2025). The effective use of portland cement as binder replacement in reactive powder-based hybrid asphalt concrete. J. Clean. Prod..

[88] Xia H., Zhao X., Wu Y., Yuan T., Song L., Yan M., Wang F., Chen H. (2020). Preparation and performance of antifreeze adhesive materials for asphalt pavement. Constr. Build. Mater..

[89] Poovaneshvaran S., Mohd Hasan M.R., Putra Jaya R. (2020). Impacts of recycled crumb rubber powder and natural rubber latex on the modified asphalt rheological behaviour, bonding, and resistance to shear. Constr. Build. Mater..

[90] Sanjaya B.G.V., Appuhamy J.M.R.S., Bandara W.M.K.R.T.W., Venkatesan S., Gravina R.J. (2025). The potential of recovering design strength of rubberized concrete using aqua-thermally treated crumb rubber at lower replacement ratios. Constr. Build. Mater..

[91] Xie J., Li S., He W., Ding Z., Lu Z., Zhao X. (2025). Molecular simulation of graft-activated crumb rubber modified asphalt: A study on high temperature performance and its interface behavior with aggregate. Surf. Interfaces.

[92] Aljarmouzi A., Dong R., Zhao M. (2025). Molecular dynamics simulation and experimental study on crumb waste rubber devulcanization using waste oils. J. Mol. Liq..

[93] Abdolahi S., Soleiman-Beigi M. (2025). Design of natural asphalt sulfamic acid (NA-NHSO_3_H) as a scalable natural asphalt-derived heterogeneous Brønsted acid to catalyze multicomponent reactions meeting green chemistry goals. Heliyon.

[94] Zakerzadeh M., Shahbodagh B., Ng J., Khalili N. (2024). The use of waste tyre rubber in Stone Mastic Asphalt mixtures: A critical review. Constr. Build. Mater..

[95] Esmaeili N., Alavi M.Z., Samadzad M. (2025). Evaluation of the impacts of polymeric fibers and modifiers on the fracture properties of asphalt mixtures. Results Eng..

[96] Wang T., Riccardi C., Jiang W. (2025). From waste to sustainable pavement: Rejuvenation of asphalt binder using waste engine oil residue and crumb rubber. Chem. Eng. J..

[97] Fernandes S., Silva H.M.R.D., Oliveira J.R.M. (2019). Mechanical, surface and environmental evaluation of stone mastic asphalt mixtures with advanced asphalt binders using waste materials. Road Mater. Pavement Des..

[98] Dong R., Zhao M., Tang N. (2019). Characterization of crumb tire rubber lightly pyrolyzed in waste cooking oil and the properties of its modified bitumen. Constr. Build. Mater..

[99] Meng Y., Chen J., Kong W., Wang Z., Lu Y., Chen P. (2024). Research on the influence of parameters on the fracture performance of the large stone asphalt mixture based on the semi-circular bending test. Constr. Build. Mater..

[100] Obaid H.A., Eltwati A., Hainin M.R., Al-Jumaili M.A., Enieb M. (2024). Modeling and design optimization of the performance of stone matrix asphalt mixtures containing low-density polyethylene and waste engine oil using the response surface methodology. Constr. Build. Mater..

[101] Oner J. (2023). Evaluation of mechanical properties for stone mastic asphalt containing textile waste. Matér. Rio Jan..

[102] Yang S., Zhu H., Li R., Yang X., Tan Q., Chen Y., Lei L. (2024). Application of functionalized graphene oxide in the preparation of crumb rubber modified asphalt with excellent storage stability. Constr. Build. Mater..

[103] Tang N., Luo M., Xue C., Liu S., Li R., Zhu H., Cheng H. (2025). Performance evaluation and mechanism investigation of aged SBS/chemically activated rubberized asphalt. Constr. Build. Mater..

[104] Li Y., Zhao C., Li R., Zhang H., He Y., Pei J., Lyu L. (2025). Dry-process reusing the waste tire rubber and plastic in asphalt: Modification mechanism and mechanical properties. Constr. Build. Mater..

[105] Hu C., Sun Z., Xi L., Tian W., Zhang H. (2024). Rheology and Phase Morphology Properties of Recycled Asphalt Modified with Waste Cooking Oil and Activated Crumb Rubber. Int. J. Pavement Res. Technol..

[106] Ferdaus R., Masri K.A., Hasan K., Jaya R.P. (2025). A comprehensive review of the utilization of alternative binding properties in the construction of asphalt pavements. Int. J. Adhes. Adhes..

[107] Hu M., Ji S., Li M., Zhu K., Sun D. (2025). Reutilization of waste-based oils for efficient recycling of aged highly polymer modified asphalt: A case study of principal component-cluster analysis. Constr. Build. Mater..

[108] Tushar Q., Santos J., Zhang G., Robert D., Giustozzi F. (2025). Recycled used cooking oil (UCO) as a rejuvenator in high content reclaimed asphalt pavement (RAP) mixes: A life cycle assessment (LCA). Sci. Total Environ..

[109] Li J., Santos J., Vargas-Farias A., Castro-Fresno D., Xiao F. (2024). Prospective LCA of valorizing end-of-life tires in asphalt mixtures with emerging pretreatment technologies of crumb rubber. Resour. Conserv. Recycl..

[110] Akhtar A.Y., Tsang H.-H. (2024). Dynamic leaching assessment of recycled polyurethane-coated tire rubber for sustainable engineering applications. Chem. Eng. J..

[111] Wang T., Shi C., Liu S., Fu C., Yang J., Liu P. (2025). Laboratory evaluation on the long-term performance of crumb rubber asphalt mixture for elastic track bed. Case Stud. Constr. Mater..

[112] Zhang F., Ma T., Rong H., Wang Z., Zhao J. (2025). A comparative study of modified and rubber asphalt pavements with semi-rigid and rigid bases based on field experiments. Constr. Build. Mater..

[113] Ortiz De Zarate F.I., Ferrero I.Z., Regenhardt S.A., Botasso H.G., Chen H., Liu G., Meyer C.I. (2025). Experimental study on the impact of organoclay surfactant loading on the microstructure and properties of crumb rubber modified asphalts. Constr. Build. Mater..

[114] Huang S., Chen H., Niu D., Ren S., Liu X. (2025). Insights into the micro-modification mechanism, thermal stability, and rheological property of SBS/TB rubber crumb modified asphalt binder. Constr. Build. Mater..

[115] Zhang T., Gao S., He Y., Liu Q., Xu S., Zhuang R., Zeng S., Yu J. (2024). Development of sustainable desulfurized waste crumb rubber modified asphalt with enhanced self-healing properties. Constr. Build. Mater..

[116] Yan Y., Guo R., Zhang S., Yang Y., Liu Z. (2025). Effect of crumb rubber depolymerisation degree on the properties of modified asphalt. Case Stud. Constr. Mater..

[117] Huang Y., Long K., Qu C., Yan C., Ai C., Zhou S. (2025). Evaluation of terminal blend rubberized asphalt incorporating high crumb rubber content. Case Stud. Constr. Mater..

[118] Zhao Z., Xiao F., Toraldo E., Crispino M., Antoniazzi A. (2025). Radiation-thermal-moisture coupling effects on viscoelasticity properties of reclaimed asphalt rejuvenated by rubberized asphalt. Constr. Build. Mater..

[119] Xie J., Zhou Y., Zhao X., Jiang Y., Huang H., Ye Q. (2025). Effects of biological desulfurization on storage stability of crumb rubber modified asphalt: Experimental analysis and molecular simulation. Constr. Build. Mater..

[120] Li D., Shen M., Chen Z., Zhang S., Yin B., Leng Z. (2025). Feasibility and performance of recycling reclaimed asphalt pavement using activated rubber pellets through semi-wet process. Constr. Build. Mater..

[121] Li H., Khair A., Han Y., Zhu T., Chen L., Lin Z., Harvey J. (2025). Investigation on high-content pretreated crumb rubber asphalt modified with polymers for sustainable and resilient pavement. Constr. Build. Mater..

[122] Zhang M., Su Q., Li G., Cao D., Yao Y., Yang S., Wang S. (2025). Enhancing Reutilization of Waste Tires and Sustainability of Environment: Analysis of the Performance and Emission Reduction Mechanism of High Content Rubber Modified Asphalt. Chem. Eng. J..

[123] Martinez-Soto A., Valdes-Vidal G., Calabi-Floody A., Avendaño-Vera C., Martínez-Toledo C. (2022). Comparison of Environmental Loads of Fibers Used in the Manufacture of Hot Mix Asphalt (HMA) and Stone Mastic Asphalt (SMA) Mixes Using a Life Cycle Assessment (LCA). Sustainability.

[124] Toghroli A., Mehrabi P., Shariati M., Trung N.T., Jahandari S., Rasekh H. (2020). Evaluating the use of recycled concrete aggregate and pozzolanic additives in fiber-reinforced pervious concrete with industrial and recycled fibers. Constr. Build. Mater..

[125] Isa M.N., Pilakoutas K., Guadagnini M., Angelakopoulos H. (2020). Mechanical performance of affordable and eco-efficient ultra-high performance concrete (UHPC) containing recycled tyre steel fibres. Constr. Build. Mater..

[126] Pi Y., Wang X., Zhu Y., Naseri A., Zarei M. (2025). Laboratory study of the effect of coal waste filler on short- and long-term fracture properties of warm mix asphalt (WMA): Towards the production of sustainable pavements. Constr. Build. Mater..

[127] Zhu Z., Xiao P., Kang A., Lou K., Kou C., Zhang Y. (2025). On enhancing the performance of modified bitumen through the synergistic mechanism of polyurethane and waste rubber powder. Energy Build..

[128] Xu G., Du M., Shen Z., Yang F., Han C. (2025). RAP Variability Intelligent Control Framework for Hot in-Plant Recycling of Asphalt Pavement. Results Eng..

[129] Shafabakhsh G., Sadeghnejad M., Mahmoudi P., Ebrahimnia R. (2025). Sustainable Nano-TiO_2_ modified asphalt with fine aggregate replacement using rubber powder. Transp. Eng..

[130] Ghazouani N., Raza A., Babeker Elhag A. (2025). Synergistic effects of SMA fibers and fly ash on the material characterization of recycled aggregate concrete. Mater. Lett..

[131] Dong F., Liu K., Yan T., Yu X., Jin Y., Li Z., Lu H. (2025). The improvement of interfacial reinforcing agent on the adhesive performance of recycled asphalt mixture: A laboratory study. Constr. Build. Mater..

[132] Guo Y., Tataranni P., Moreno-Navarro F., Martínez R.T., Sangiorgi C. (2025). Use of engineered pellets containing E-cigarette butts and a recycling agent for stone mastic asphalt mixtures incorporating recycled asphalt. Constr. Build. Mater..

[133] Jiang D., Cao Z., Gong G., Wang C., Gao Y. (2025). VOCs inhibited asphalt mixtures for green pavement: Emission reduction behavior, environmental health impact and road performance. J. Clean. Prod..

